# A prospective non-randomized interventional study of dynamic changes of L3SMI in patients with gastric cancer undergoing ambulatory chemotherapy with active nutrition combined with exercise

**DOI:** 10.3389/fonc.2025.1620115

**Published:** 2025-08-13

**Authors:** Shu-Rong Zhao, Xiao-Rong Li, Zhen-Hao Li, Xiao-Juan Ren, Ji-Xiang Hou, Jun Zhao, Gao-Wa Jin, Quan-Fu Li

**Affiliations:** 1 Ordos School of Clinical Medicine, Inner Mongolia Medical University, Ordos, China; 2 Department of Medical Oncology, Ordos Central Hospital, Ordos, China; 3 The First School of Clinical Medicine, School of Information and Engineering, WenZhou Medical University, Wenzhou, China

**Keywords:** L3 skeletal muscle index, nutrition intervention, exercise intervention, ambulatory chemotherapy, sarcopenia

## Abstract

**Objective:**

To compare the effects of combined active nutrition and exercise intervention based on a standard regimen on the dynamic changes in the L3 skeletal muscle index (L3SMI) of patients with gastric cancer receiving SOX/XELOX ambulatory chemotherapy, and explore its relationship with chemotherapy-related adverse reactions.

**Methods:**

This prospective non-randomized interventional study was conducted on patients with gastric cancer who received adjuvant or first-line SOX/XELOX ambulatory chemotherapy at Ordos Central Hospital, China, from June 2021 to December 2024. Patients receiving active nutrition and exercise intervention were defined as the intervention group, and those receiving conventional standard treatment were the control group. Compare the differences in each indicator between the two groups at the two time points before and after the intervention. Nutritional intake was assessed using the Brief Conghua scale. Nutritional management was based on the five-step nutrition program recommended in the Chinese Cancer Nutrition Treatment Guidelines 2020. The exercise program was based on FITT(frequency, intensity, time, and type) mode. WeChat was used to provide patients with education and technical guidance. Neutropenia was graded according to the Common Terminology Criteria for Adverse Events version 5.0.

**Results:**

Dynamic follow-up was completed in 77 patients, including 39 in the intervention group and 38 in the control group. The intervention group showed a positive change in the L3SMI compared to that in the control group (*P* = 0.0032). The SMI remained stable or increased in 64.1% (25/39) of patients in the intervention group and 31.6% (12/38) in the control group. The incidence of grade ≥3 neutropenia in the intervention group was lower than that in the control group (*P* = 0.0011).

**Conclusion:**

Active nutrition and exercise intervention can improve L3SMI and reduce the incidence of grade ≥3 neutropenia in patients with gastric cancer receiving SOX/XELOX ambulatory chemotherapy.

## 1 Introduction

The Global Cancer Statistics 2022 has identified gastric cancer as the fifth most common type of cancer worldwide, both in terms of incidence and mortality. It is reported that 968,000 new cases of gastric cancer are diagnosed every year (1). An analysis of the incidence and deaths resulting from malignant tumors in China in 2022, released in 2024, revealed that gastric cancer ranks fifth in the country in terms of incidence, with an estimated total number of annual cases reaching 359,000, and third in terms of deaths, with an annual mortality rate of 260,000 (2). As gastric cancer impacts the physiological functions of the digestive system, it adversely affects nutrient uptake in patients, which leads to various adverse effects in patients. It has been reported that more than 50% of patients newly diagnosed with malignant tumors also have sarcopenia (3).

Huang et al. from Wenzhou Medical University, China, used the L3 skeletal muscle index (L3SMI), measured using CT, for diagnosing sarcopenia and predicting postoperative complications and long-term survival of patients with gastric cancer undergoing radical gastrectomy. They showed that the diagnosis of sarcopenia with the help of CT was significantly better than using nutritional screening tools for predicting postoperative complications and survival of patients with gastric cancer (4). CT, in recent years, has been increasingly regarded as the gold standard for diagnosing sarcopenia. A 2019 meta-analysis of 39 studies (8402 patients) evaluated the body composition of patients with gastric cancer. Of these 39 studies, 26 used CT (5). In a previous study, we utilized the diagnostic criteria of gastric cancer sarcopenia in patients from Ordos (Northern China) and showed that more than 50% of patients with gastric cancer have sarcopenia (6). Recent studies have identified perioperative sarcopenia as an independent prognostic factor for the overall survival (OS) and cancer-specific survival (CSS) of patients with gastric cancer (7). A meta-analysis showed that the L3SMI in patients with gastric cancer significantly decreased during radiotherapy and chemotherapy, which suggests higher chemotherapy toxicity and adverse prognosis in such patients (8).

The Asian Working Group for Sarcopenia (AWGS) defines sarcopenia as an age-related loss of muscle mass accompanied by a decline in muscle strength and/or physical function (9). According to this definition, the management of sarcopenia requires interdisciplinary intervention. A nutritional survey on patients with cancer, published in 2023, showed that the patients with gastric cancer at the time of diagnosis had an energy intake of 18.4 kcal/kg/day and a protein intake of 0.8 g/kg/day, which were significantly lower than the minimum recommended target values (25 kcal/kg/day and 1.2 g/kg/day, respectively) (10). Similar results were reported by Minghua et al., who conducted a survey on the nutritional status of patients with cancer in China. Hence, it is clear that cancer patients in China have inadequate energy and protein intake (11). According to the European Society for Clinical Nutrition and Metabolism (ESPEN) guidelines, nutritional intervention for cancer patients should aim at maintaining or increasing muscle mass. Many studies suggest that active nutritional intervention after gastric cancer surgery can significantly increase energy intake and improve nutritional status (12, 13). Our team previously published a study on dynamic changes in the L3SMI during prospective active nutritional interventions and reported that the L3SMI remained stable or increased in 57.9% of patients with digestive tract tumors. Notably, the rate of decrease in the L3SMI was significantly reduced in these patients (14).

Exercise can reduce the incidence of cancer, alleviate tumor symptoms, decrease the number and extent of adverse reactions to anti-cancer treatment, and improve the efficacy of anti-cancer drugs (15). A recently published systematic review on the effects of resistance training on skeletal muscle in cancer patients suggested that resistance training has a positive effect on the overall quality and mass of skeletal muscles. Resistance training significantly increased the trunk and limb muscle mass, muscle strength (61–68%), and physical function compared to those in controls (16). A meta-analysis conducted by Liu et al. on 21 randomized controlled clinical studies that compared the effects of exercise, with or without nutritional interventions, demonstrated that a comprehensive approach combining exercise and dietary advice is more effective than exercise alone in improving physical function among older survivors of cancer. Only five articles were included in this meta-analysis regarding the effects of a combined intervention comprising nutrition and exercise. However, very few studies have investigated the effects of muscle quantity on cancer. More clinical studies are needed to explore the effect of combined exercise and nutrition intervention on sarcopenia (17).

From the perspective of public health, sarcopenia significantly increases the medical economic burden. The medical cost of hospitalized patients with sarcopenia can be more than five times that of non-sarcopenia patients (18). In China, the SOX/XELOX program is mainly administered through the ambulatory chemotherapy mode to patients with gastric cancer. The Consensus of Experts on the Daytime Diagnosis and Treatment of Malignant Tumors in China pointed out that since the time of patients receiving daytime diagnosis and treatment is significantly shortened in hospital, in order to ensure the medical safety of patients, follow-up should be carried out in accordance with the principle of whole-process management (19). The present prospective interventional study aims to compare the effects of combined active nutrition and exercise intervention on dynamic changes in L3SMI in patients with gastric cancer receiving SOX/XELOX ambulatory chemotherapy based on a standard regimen. This study was initiated during the COVID-19 pandemic. Due to severe shortages in research personnel, only control group participants could be enrolled in the initial phase, while intervention group participants were recruited after the pandemic subsided. Consequently, randomized allocation was not feasible.

## 2 Methods

### 2.1 Participants and setting

**Figure 1:**
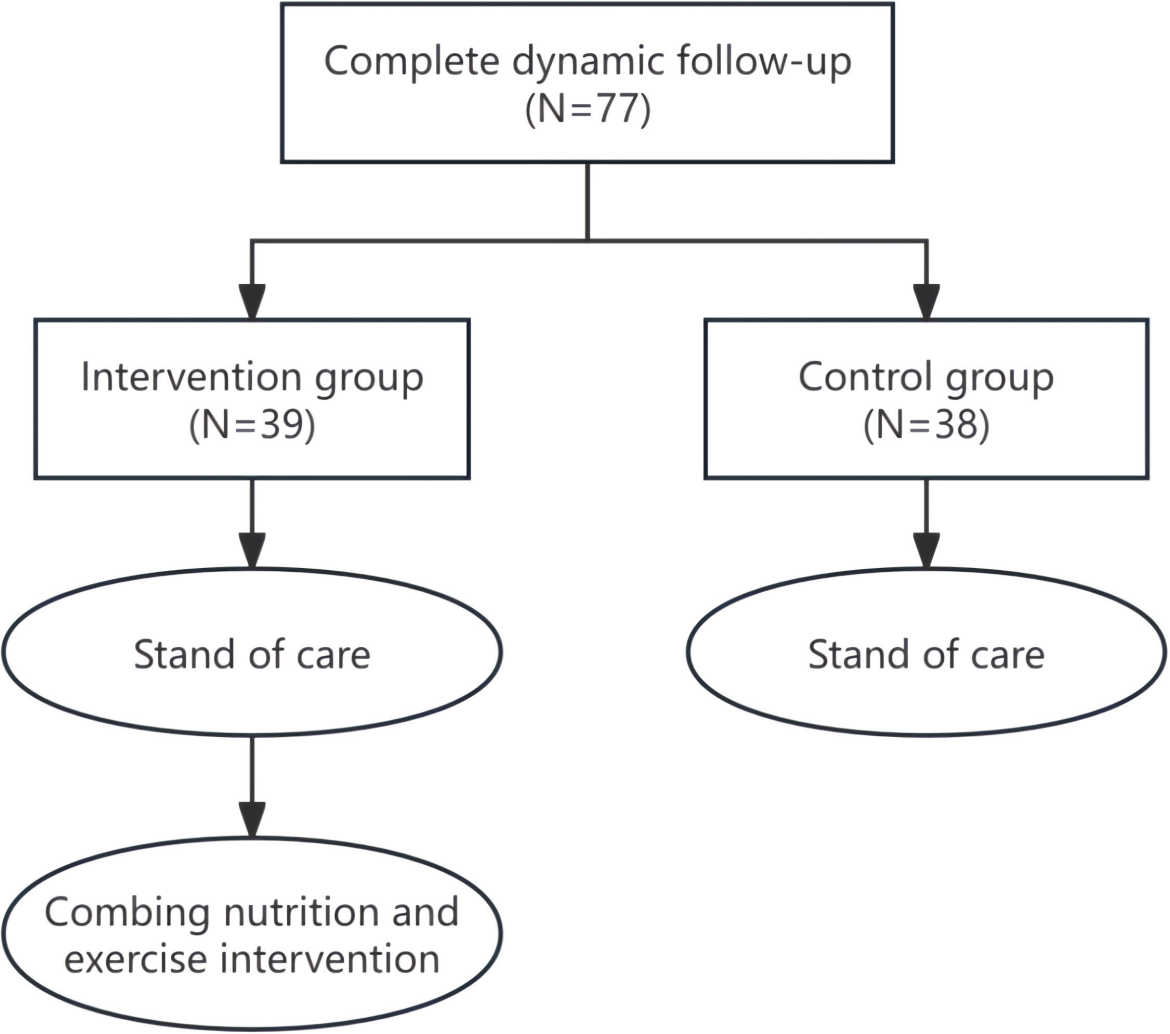
Test flowchart.

This is a prospective non-randomized interventional study. In total, 77 patients with gastric cancer who received adjuvant or first-line ambulatory chemotherapy with SOX/XELOX regimens at the Department of Medical Oncology, Ordos Central Hospital, Inner Mongolia, Northern China, from June 2021 to December 2023 were included. The oxaliplatin dose intensity was 130 mg/m² on day 1. Tegafur (S-1) (40 mg/m²) or capecitabine (1000 mg/m²) was administered on days 1–14. Each cycle lasted 21 days. Because of the impact of the COVID-19 pandemic, patients enrolled in the early phase of the study received standard treatment, while those enrolled in the later phase also received active nutrition and exercise interventions along with in-hospital patient education and out-of-hospital initiatives using the WeChat platform, a mobile phone application popular in China. Patients who received combined active nutrition and exercise intervention were placed in the intervention group, and those who received conventional standard treatment were identified as the control group (**Figure 1**). Dynamic changes in the L3SMI, the incidence of neutropenia, changes in the Nutritional Risk Screening 2002 (NRS-2002) and Patient-Generated Subjective Global Assessment (PG-SGA) scores, and bioelectrical impedance analysis (BIA) indicators of both the intervention and control groups after 12 weeks of chemotherapy were compared. To control for potential confounding bias, this study compared baseline characteristics between the intervention and control groups, ensured consistent treatment protocols and measurement methods across all patients, and employed sensitivity analyses to further adjust for potential confounding factors. This study was approved by the Medical Ethics Committee of Ordos Central Hospital (2021–013 and 2023-21). All subjects provided written informed consent. The study has been registered at the Chinese Clinical Trial Center (ChiCTR2200056758 and ChiCTR2300071748).

#### 2.1.1 Inclusion criteria

The following inclusion criteria were employed:

- Patients aged ≥18 years with pathologically confirmed gastric malignancy were enrolled, provided they fulfilled the following conditions:
  ▪ received one–two cycles of SOX/XELOX chemotherapy
  ▪ have undergone at least four consecutive cycles of ambulatory chemotherapy at the Department of Oncology, Ordos Central Hospital;
- Patients with a Karnofsky Performance Status (KPS) score of ≥70;
- Patients without any severe cardiopulmonary disease that can affect the ability to tolerate the prescribed exercise regimen;
- Patients who can independently operate the WeChat application on a mobile device to communicate with the medical team;
- Patients eligible for regular BIA and computed tomography (CT) follow-up;
- Patients with Hb ≥ 90 g/L;
- Patients without any anemia-induced movement disorders;
- Patients who provided informed consent;
- Patients who adhered to the scheduled treatment plan till its completion.

#### 2.1.2 Exclusion criteria

The exclusion criteria were as follows:

- Patients who changed their treatment method or chemotherapy regimen;
- Patients with gastrointestinal or intestinal obstruction symptoms that prevent oral nutrition intake;
- Patients with severe consumptive diseases, such as diabetes and hyperthyroidism;
- Patients on long-term hormone therapy;
- Patients with arterial or venous thromboembolic complications that contraindicate physical activity;
- Patients with scoliosis or other physical deformities affecting body composition assessment;
- Patients with gastrointestinal stomas or other conditions affecting moderate-intensity physical activity;
- Patients with the metastasis of brain or bone.

### 2.2 Intervention

All enrolled patients received four cycles of the XELOX/SOX ambulatory chemotherapy. The intervention group received team-based patient education that integrates both nutrition and exercise during each chemotherapy cycle, with the following four education timepoints: T1, day 1 of ambulatory chemotherapy administration; T2, day 4 of chemotherapy; T3, day 10 of chemotherapy; and T4, day 17 of chemotherapy. At the T1 time point, a full-time research assistant explained the combined intervention program (comprising active nutrition along with exercise) and its potential benefits to the patients of the active intervention group for 30 min. The family members and supporters of the patients were encouraged to participate in each face-to-face session to maximize the effectiveness and safety of the intervention. From T2 to T4, the patients and family members were provided with information related to nutrition and exercise, technical guidance, compliance review, and personalized intervention optimization through outpatient clinics or the WeChat platform. If a patient failed to complete ≥50% of the prescribed exercise intervention volume during a chemotherapy cycle despite being actively provided with education and intervention, they were removed from the group. The nutrition and exercise interventions were supervised by dedicated nutrition research assistants, while the outcome variable measurements and analyses were independently conducted by another researcher in team to ensure objectivity.

#### 2.2.1 Nutrition intervention

The Brief Minghua Scale was used to assess the nutritional intake of the patients (20). Clinical nutrition management refers to the five-step nutrition program recommended by the China Cancer Nutrition Treatment Guidelines 2020 (21). Nutritional assessment of both the intervention and control groups was carried out based on the NRS-2002 and PG-SGA scales. As per the energy and protein intake standards, the patients were required to meet at least 70% of their daily intake target (including diet + enteral nutrition). Considering that body mass is a core assessment criterion in the GLIM global consensus standards for malnutrition (22). Specific requirements included a total energy demand of 25–30 kcal/kg/day (calculated based on the patient’s ideal body mass according to their stature) and a total protein requirement of 1.2–2.0 g/kg/day (also based on the ideal body mass). Patients in the intervention group were advised to consume one egg and 250 mL of pure milk per day (milk replaced with yoghurt for people with lactose intolerance) to maintain an adequate protein intake. The five steps of the nutritional support pathway included the following: ① diet + nutrition education, ② diet + oral nutrition supplement (ONS), ③ total enteral nutrition support (TEN), ④ partial enteral nutrition + partial parenteral nutrition support (PPN), and ⑤ total parenteral nutrition support (TPN). When any step failed to meet 60% of the target requirements for more than 3–5 days, the next step of the nutritional treatment was selected. (To ensure accessibility, the main components of the two medical insurance-reimbursed compound nutritional formulations are: For ONS: Total Protein and Fibre, indicated for Tumor patients (TPF-T) (Fresenius Kabi SSPC) containing protein (29.25 g), fat (36 g), and carbohydrate (52 g), providing 2730 kJ (650 kcal) with electrolytes. For TPN: Fat Emulsion, Amino Acids (17) and Glucose (11%) Injection (Fresenius Kabi AB) containing glucose 11% (885 mL), Vamin 18 Novum (300 mL), and Intralipid 20% (255 mL), with a total energy content of 1000 kcal and electrolytes.) The following were used as indications of enteral nutrition: moderate-to-severe dysphagia, >5% body mass loss within one month, body mass index (BMI)< 18.5 kg/m^2^, PG-SGA ≥ 4, food intake< 60% of the normal requirement for more than 3–5 days, with poor digestion and absorption function.

As part of the enteral nutrition supplement, 400–600 mL of the ONS was given daily. The nutritional regimen was adjusted by a nutritionist at the four educational time-points, based on the nutritional status (especially body mass), dysphagia, swallowing pain, food intake, and diet structure of the patients. If the enteral nutrition supplement did not meet the current nutritional requirements of the patients, the patients were recommended hospitalization so that they could be provided with better nutritional treatment.

#### 2.2.2 Exercise program

We designed an exercise program for the patients based on the frequency, intensity, time, and type (FITT) model developed by the American College of Sports Medicine Roundtable on Exercise Guidelines for Cancer Survivors (23). The FITT model recommends that after undergoing ambulatory chemotherapy, patients should engage in cardiorespiratory exercise for an average of no less than 150 minutes per week and resistance exercise for no less than 2 days per week, with the intensity maintained at low-to-moderate levels (Cardiorespiratory exercise:low intensity: able to speak or sing calmly and continuously during physical activity, with heart rate and breathing remaining essentially steady; moderate intensity: able to continue speaking but unable to sing, with increased heart rate and breathing frequency but not rapid; high intensity: unable to speak continuously, only able to utter short phrases, with significantly elevated heart rate and rapid breathing. Resistance training:low intensity: 30%-49% 1-RM; moderate intensity: 50%-69% 1-RM; high intensity: 70%-89% 1-RM.1-RM (1 Repetition Maximum) refers to the maximum load that can be lifted once through a full range of motion with proper form and technique). During the first week after chemotherapy, the exercise regimen can be adjusted to no less than 150 min of low-intensity cardiorespiratory exercise plus 1 day of resistance exercise, followed by maintaining the standard regimen thereafter. It is advised to perform a 5-min warm-up activity before exercising.

##### 2.2.2.1 Pre-exercise assessment

It is recommended that an exercise risk assessment of the patients be conducted based on their own conditions before exercise and warm-up before starting. If increased physical activity leads to exacerbated fatigue or other adverse reactions, the FITT parameters of the exercise prescription should be reduced to a level tolerable for the patient. It is recommended to measure the heart rate and blood pressure of the patient before exercise, with weekly routine blood tests.

##### 2.2.2.2 Cardiorespiratory exercise

The average time of cardiorespiratory exercise should not be less than 150 min per week. The main form of exercise should be walking, although other cardiorespiratory activities are also allowed. The following scheme was recommended, which we used in combination with WeChat (24):

- If the average number of daily steps of patients was less than 5000, the target number of steps was set at 5000.
- If the average number of daily steps of patients was between 5001 and 7999, the target number of steps was set as the average number of steps plus 2000 to a maximum of 8000 steps.
- If the average number of daily steps of patients was ≥8000, the patients were encouraged to maintain their current level.

##### 2.2.2.3 Resistance training

Resistance training should be performed at least two days per week, with two sets executed daily. Each set should include at least 10 repetitions of resistance exercises targeting major muscle groups in the chest, shoulders, arms, back, abdomen, and legs. The following exercises were recommended: chest press, bicep curls, tricep extensions, leg press, leg curls, leg extensions, sit-ups, hip abduction, hip adduction, step-ups, and sit-to-stand transitions. Patients can select at least two of these activities based on their conditions. During the initial period, the patients should be guided to complete 10 repetitions of each movement without any added load to ensure proper adaptation to resistance training. If the participant adapts well, the load should be gradually augmented, followed by progressive increases in repetitions. However, if increased physical activity leads to fatigue or adverse reactions, the FITT principles of the exercise prescription should be reduced to a level the patient can tolerate.

### 2.3 Objectives

To compare the effects of combined active nutrition and exercise intervention based on a standard regimen on the dynamic changes in the L3 skeletal muscle index (L3SMI) of patients with gastric cancer receiving SOX/XELOX ambulatory chemotherapy, and explore its relationship with chemotherapy-related adverse reactions.

### 2.4 Outcomes

#### 2.4.1 Primary outcomes

To compare the dynamic changes of the L3SMI between the intervention group and the control group after 12 weeks of chemotherapy.

#### 2.4.2 Secondary outcomes

To compare the incidence of neutropenia between the two groups, as well as changes in their NRS 2002 scores, PG-SGA scores, and bioelectrical impedance parameters.

### 2.5 Sample size

Sample sizes of 38 per group provide 90% power (two-sided α=0.05) to reject the null hypothesis of equal means when the population mean difference is 3.0 (with a standard deviation of 4.0 in both groups), as calculated by a two-sample t-test assuming equal variance.

### 2.6 Data collection

#### 2.6.1 General information collection

The general information of all study subjects was collected (including their names, sex, ages, places of origin, contact information, disease type, pathological stage, and initial chemotherapy regimen) during their first hospitalization.

#### 2.6.2 Stature and body mass measurement

The stature and body mass of the patients were measured using standard methods during each hospitalization. The BMI and body surface area (BSA) of the patients were calculated using the following formulas:


$$\text{BMI=Body mass}\left(\text{kg}\right){\text{/Stature}}^{2}\left({\text{m}}^{2}\right)$$

$$\text{BSA }\left({\text{m}}^{2}\right)\text{=[Stature}\left(\text{cm}\right)\text{+Body mass}\left(\text{kg}\right)-60]/100$$

#### 2.6.3 Body composition measurement

The body composition was determined through the following tests. The data were recorded at baseline and after the completion of four cycles of chemotherapy.

- NRS 2002 and PG-SGA assessment: All subjects were assessed by a professional within 24 h of admission.
- BIA determination: The physical composition (including body fat mass, skeletal muscle mass, and body fat percentage) of all enrolled patients was measured by using a multifrequency bioelectrical impedance analyzer (DBA-550). To use the bioelectrical impedance method to measure the body composition, the room temperature should be maintained at an ambient level (20–25°C), the measurement should be taken on an empty stomach, and both the bladder and bowel should be emptied before conducting measurements, and no measurements should be taken after an exercise shower or during a woman’s menstrual period. Fat-free body mass index (FFMI) was calculated as follows:

$$\begin{array}{c} \text{FFMI }=\;\left(1\;-\;\text{percentage of body fat}\right)\\ \times \text{body mass}\left(\text{kg}\right)÷\text{stature}\left({\text{m}}^{2}\right) \end{array}$$

FFMI low standard: male ≤17.4 kg/m^2^, female ≤ 15.0 kg/m^2^ (21)

- Abdominal CT scan: Before starting the chemotherapy, the abdominal CT scan of the patients was conducted on the continuous layer of the lumbar 3 vertebrae with -29 to 150 HU units and 5-mm thickness. The muscle area was analyzed using the images of two continuous transverse sections of the same sequence of the lumbar 3 vertebrae. The areas of the skeletal muscles (including the psoas major, erects spinalis, quadratus lumbois, and transversoas muscles) were calculated using the radiotherapy treatment planning system (TPS) software XIO. The cross-sectional areas of skeletal muscles (including the psoas major, erector spinae, quadratus lumborum, transversus abdominis, internal oblique muscles, and external oblique muscles) were measured and summed. The L3SMI was calculated as follows:

$$\text{SMI }\left({\text{cm}}^{2}/{\text{m}}^{2}\right)= L3 skeletal muscle area\left({\text{cm}}^{2}\right)\text{/Statur}{\text{e}}^{2}\left({\text{m}}^{2}\right)$$

The lean body mass (LBM) was derived using the formula: (25)


$$\text{LBM }\left(\text{kg}\right)\mathrm{= 0}.3\;\times \;\left[\mathrm{L3 skeletal muscle area}\left({\text{cm}}^{2}\right)\right]\mathrm{+ 6}.06$$

#### 2.6.4 Laboratory tests and adverse reaction monitoring

Fasting venous blood of the patients was collected within 24 h of admission at baseline and 12 weeks post-treatment for testing. Their neutrophil counts were recorded, and the adverse reactions were graded and documented based on CTCAE version 5.0 (26).

### 2.7 Statistical methods

Continuous variables subject to normal distribution were described in the form of mean ± standard deviation (SD), while those not subject to normal distribution were described as the median (interquartile range). Categorical variables were described as frequency (percentage). The *t*-test was used to compare the difference between the two groups for continuous variables with normal distribution. The Wilcoxon rank-sum test was used for continuous variables without normal distribution. The categorical variables were tested by the Chi-square test or Fisher’s exact test.

Analysis of the covariance model (ANCOVA) was used to compare differences regarding L3SMI and other indicators between the intervention group and the control group during chemotherapy. The change in the values of L3SMI and other indicators from baseline was used as the dependent variable. The group was used as the fixed effect. The baseline value of L3SMI and other indicators was used as the covariable in the ANCOVA model. In sensitivity analysis, the baseline values of L3SMI and other indicators with statistically significant differences between the groups were used as covariates. The least squares mean changes from baseline for L3SMI and other indicators for the two groups were calculated, along with their standard errors. Additionally, the mean of the least squares mean difference between the groups and its 95% confidence interval (95% CI) were computed.

The logistic regression model was used to analyze the difference in chemotherapy-related neutropenia between the intervention and control groups. The dependent variable was grade ≥3 chemotherapy-related neutropenia in the follow-up period and group as the fixed effect, while the covariate was chemotherapy-related neutropenia of grade ≥3 at baseline. The odds ratio (OR) and 95% CI were calculated. In addition, a logistic regression model was used to analyze the type of treatment and chemotherapy-related neutropenia, with chemotherapy-related neutropenia of grade ≥3 as the dependent variable, treatment type as the fixed effect, the group as the baseline covariate, and group and baseline chemotherapy-related neutropenia of grade ≥3 as the follow-up covariate.

All of the above analyses were performed using SAS software version 9.4. All statistical tests were bilateral, and a *P*-value< 0.05 was considered statistically significant.

## 3 Results

### 3.1 Baseline characteristics of patients

From June 2021 to December 2024, 77 subjects completed the dynamic follow

-up of L3SMI in four consecutive cycles of XELOX/SOX ambulatory chemotherapy. The intervention group included 39 patients who received combined intervention comprising active nutrition along with exercise, while the control group had 38 patients who received standard treatment. Eighteen patients failed to reach the 50% physical activity threshold and were excluded.

The clinical baseline characteristics of the 77 patients are listed in **Table 1**. Men accounted for 81% of these patients, with a median age of 63.17 ± 10.35 years, consistent with the epidemiological characteristics of patients with gastric cancer reported in 2022 (27). The China Cancer Nutrition Treatment Guidelines 2020 were used to classify patients based on their BMI values: BMI< 18.5, underweight (malnutrition); BMI< 18.5–23.9, normal; BMI< 24–27.9, overweight; and BMI ≥ 28, obese (21). Among the 77 patients, 68% (52/77) had normal BMI, while 17% (13/77) were overweight or obese, indicating that most of the newly diagnosed or post

**Table 1:** Baseline characteristics (N=77).

Variables	Intervention group (N=39)	Control group (N=38)	P*
Sex			0.4195
Male	30 (76.92%)	32 (84.21%)	
Female	9 (23.08%)	6 (15.79%)	
Age, years	64.31±10.64	62.00±10.05	0.3313
Treatment			0.2668
Adjuvant	23 (58.97%)	27 (71.05%)	
Palliative	16 (41.03%)	11 (28.95%)	
BMI (kg/m^2^)			0.7722
Underweight	5 (12.82%)	7 (18.42%)	
Normal	26 (66.67%)	26 (68.42%)	
Overweight	6 (15.38%)	3 (7.89%)	
Obesity	2 (5.13%)	2 (5.26%)	
BSA (m^2^)	1.71±0.16	1.67±0.16	0.2493
FP (%)	19.60±8.33	18.82±9.53	0.7058
FFMI	17.60±1.94	17.06±1.75	0.2074
L3SMI (cm^2^/m^2^)	29.15±7.13	36.04±7.33	<0.0001
Grade≥3 neutropenia	3 (7.69%)	6 (15.79%)	0.3100

*P-**value**: Chi-square test or Fishier exact test was used for categorical variables, and T-test or Wilcoxon rank sum test was used for continuous variables.

BMI, body mass index; BSA, body surface area; FP, fat percentage; FFMI, fat-free body mass index; PG-SGA, Patient-Generated Subjective Global Assessment.

NRS -2002, Nutritional Risk Screening Scale; L3SMI, L3 skeletal muscle index.

-operative patients with gastric cancer maintained normal BMI levels. As shown in **Table 1**, the baseline differences in NRS-2002, PG-SGA, and L3SMI between the two groups were statistically significant. When the Asian diagnostic criteria for sarcopenia (men, L3SMI< 36 cm²/m²; women, L3SMI< 29 cm²/m²) were used (28), we found that the intervention group had a higher proportion of patients with sarcopenia than the control group (82.05% (32/39) vs. 47% (18/38)). However, the control group had significantly higher L3SMI values, suggesting that patients with sarcopenia were more likely to receive active nutritional and exercise interventions.

### 3.2 Primary outcome

#### 3.2.1 L3SMI

The patients were categorized into three groups based on changes in their SMIs before and after chemotherapy: those with an SMI decrease more than 2% were considered to suffer from skeletal muscle loss; those with an SMI increase more than 2% were identified to have skeletal muscle gain; and those with changes between these two thresholds were regarded to have stable skeletal muscles (29). The proportion of patients in the intervention group with a stable or increased L3SMI was significantly higher than that in the control group (64.1% (25/39) vs. 31.6% (12/38)).

**Table 2.1** compares differences in the changes of L3SMI (ΔL3SMI) between the intervention group and the control group. Irrespective of whether the differences in the ΔL3SMI of the two groups before and after the intervention were analyzed through the arithmetic mean or least squares method, we found that the intervention group showed positive ΔL3SMI, while the control group exhibited negative ΔL3SMI. The L3 skeletal muscle area (L3SMA) and LBM were determined using CT-measured indicators. The intervention group demonstrated positive changes in both metrics, whereas the control group showed negative changes in both.

**Table 2.1:** Changes in muscle-related indexes measured by CT (N=77).

Variables	Mean±SD		LsMean±SD		Difference between groups	
	Intervention group (N=39)	Control group (N=38)	Intervention group (N=39)	Control group (N=38)	LsMean (95%CI)	*P*
ΔL3SMI	0.86±3.77	-2.64±4.37	0.69±0.69	-2.46±0.70	3.15 (1.09-5.21)	0.0032
ΔL3SMA	2.41±10.67	-8.38±12.56	1.67±1.94	-7.62±1.97	9.29 (3.54-15.04)	0.0019
ΔLBM	0.72±3.20	-2.51±3.99	0.50±0.58	-2.29±0.59	2.79 (1.06-4.51)	0.0019

L3SMI, L3 skeletal muscle index; L3SMA, L3 skeletal muscle area; LBM, lean body mass.

Sensitivity analyses were performed on the three aforementioned metrics to control for various confounding factors. The conclusions derived from **Table 2.2** are consistent with the results presented in **Table 2.1**.

**Table 2.2:** Sensitivity analysis based on changes in muscle-related indicators measured by CT (N=77).

Variables	LsMean±SD		Difference between groups	
	Intervention group (N=39)	Control group (N=38)	LsMean (95%CI)	*P*
ΔL3SMI	0.95±0.73	-2.73±0.74	3.68 (1.41-5.94·)	0.0018
ΔL3SMA	2.88±2.06	-8.86±2.10	11.74 (5.35-18.12)	0.0005
ΔLBM	0.86±0.62	-2.66±0.63	3.52 (1.61-5.44)	0.0005

L3SMI, L3 skeletal muscle index; L3SMA, L3 skeletal muscle area; LBM, lean body mass.

**Figure 2:**
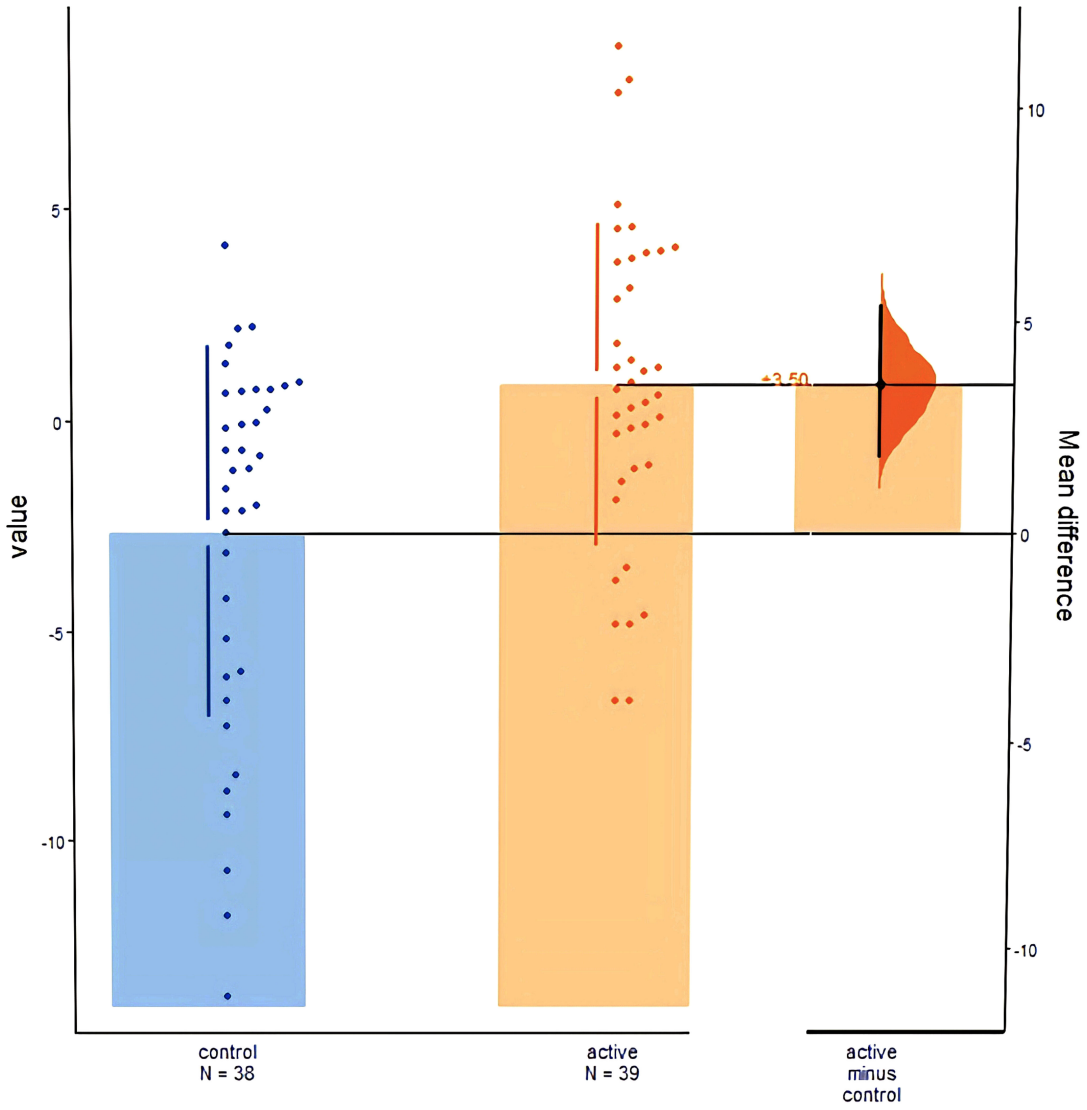
L3SMI data distribution and differences.

**Figure 2** clearly illustrates the L3SMI distribution and longitudinal changes in the intervention versus control groups, reconfirming the statistically significant intergroup difference.

### 3.3 Secondary outcomes

#### 3.3.1 Chemotherapy-associated neutropenia

The incidence of chemotherapy-related neutropenia was recorded according to the National Institutes of Health Standard V.5.0 for General Terms of Adverse Events. As shown in **Table 3**, the intervention group exhibited a significantly lower incidence of grade ≥3 neutropenia during follow-up compared to that exhibited by the control group, indicating that active nutrition combined with exercise intervention effectively reduces the occurrence of neutropenia during chemotherapy.

**Table 3:** Comparison of chemotherapy-associated neutropenia in two groups (N=77).

	Intervention group (N=39)	Control group (N=38)	OR (95% CI)	*P*
Grade ≥3 neutropenia	6 (15.38)	21 (55.26)	0.13 (0.04, 0.44)	0.0011

#### 3.3.2 Comparison of dynamic changes of NRS-2002 and PG-SGA scales

**Table 4** compares differences in the changes of NRS-2002 and PG-SGA scores of the two groups before and after the intervention; lower scores indicate a lower nutritional risk. Although the intervention group exhibited lower scores than the control group, the differences were not statistically significant (*P* = 0.9135; *P* = 0.6359).

**Table 4:** Changes in NRS-2002 nutritional risk screening form, PG-SGA nutritional assessment form (N=77).

Variables	Mean±SD		LsMean±SD		Difference between groups	
	Intervention group (N=39)	Control group (N=38)	Intervention group (N=39)	Control group (N=38)	LsMean (95%CI)	*P*
ΔNRS-2002	-0.13±0.98	-0.97±2.05	-0.53±0.22	-0.56±0.22	-0.04 (-0.61,0.68)	0.9135
ΔPG-SGA	-0.64±3.57	-1.55±3.98	-1.25±0.48	-0.92±0.49	-0.33 (-1.72,1.06)	0.6539

NRS-2002, Nutritional Risk Screening 2002; PG-SGA, Patient-Generated Subjective Global Assessment.

#### 3.3.3 Comparison of dynamic changes of bioelectrical impedance index

**Table 5** describes the FFMI and fat percentage (FP) as measured by BIA. The intervention group showed greater increases in ΔFFMI and ΔFP compared to that demonstrated by the control group, suggesting that BIA may have lower sensitivity than CT in detecting body composition. Additionally, the intervention group exhibited higher ΔBMI and ΔBSA than the control group.

**Table 5:** Changes in the related indexes of physical composition measured by BIA (N=77).

Variables	Mean±SD		LsMean±SD		Difference between groups	
	Intervention group (N=39)	Control group (N=38)	Intervention group (N=39)	Control group (N=38)	LsMean (95%CI)	*P*
ΔFP	-1.19±4.98	-1.91±4.27	-1.13±0.70	-1.98±0.71	0.85 (-1.15-2.85)	0.3991
ΔFFMI	0.09±1.23	0.18±0.75	0.14±0.16	0.13±0.16	0.01 (-0.43-0.46)	0.9494
ΔBMI	-0.16±1.20	-0.84±1.34	-0.11±0.20	-0.89±0.20	0.78 (0.23-1.34)	0.0065
ΔBSA	-0.005±0.033	-0.024±0.041	-0.004±0.006	-0.026±0.006	0.02 (0.006-0.038)	0.0079

BMI, body mass index; BSA, body surface area; FP, fat percentage; FFMI, fat-free body mass index.

#### 3.3.4 Association between chemotherapy type and grade ≥3 chemotherapy-induced neutropenia

As shown in **Table 6**, the incidence of grade ≥3 chemotherapy-related neutropenia was significantly lower in patients receiving adjuvant therapy compared to those receiving palliative therapy (*P* = 0.0308), indicating a correlation between the treatment type at baseline and the occurrence of grade ≥3 neutropenia.

**Table 6:** Correlation of treatment type with ≥ grade 3 chemotherapy-associated neutropenia (N=77).

	Adjuvant treatment group (N=50)	Palliative treatment group (N=27)	*P*
Baseline	3 (6.00)	6 (22.22)	0.0308
Follow-up	17 (34.00)	10 (37.04)	0.9333

## 4 Discussion

This prospective interventional study evaluated the effects of combined nutrition and exercise intervention on dynamic changes in the L3SMI among gastric cancer patients receiving SOX/XELOX chemotherapy. The primary endpoint of the study was L3SMI. Under the combined intervention involving active nutrition and exercise, a significantly greater improvement in ΔL3SMI was noted in the intervention group than that demonstrated by the control group (*P=*0.0032). The sensitivity analysis results also confirmed that the intervention group demonstrated a significantly greater improvement in ΔL3SMI than that shown by the control group. Of the 77 enrolled patients, 64.1% (25/39) of patients in the intervention group maintained or increased their SMI, compared with 31.6% (12/38) of the patients in the control group. Furthermore, the incidence of grade ≥3 neutropenia was significantly lower in the intervention group compared to controls (*P*=0.0011).

A systematic review and meta-analysis of 38 studies involving 7,843 patients with solid tumors revealed that 27.7% of these patients had reduced muscle cross-sectional area, and that sarcopenia was associated with decreased overall survival (hazard ratio (HR): 1.44, 95% CI: 1.32–1.56) (30). According to the international diagnostic threshold for sarcopenia, as many as 47% of patients with gastric cancer worldwide have sarcopenia (31), although the international diagnostic threshold is significantly higher than the Asian threshold. Notably, the incidence of sarcopenia among Chinese patients with gastric cancer is higher. Therefore, the development of methods for preventing and treating sarcopenia is an important clinical topic. The nutrition and exercise combination method in patients with cancer is the current research focus. However, the current literature base on integrated nutrition and exercise programs lacks strong evidence (30). To our knowledge, this is the first prospective clinical study on the combination method comprising nutrition and exercise in patients with gastric cancer undergoing chemotherapy in China.

This study follows a prospective intervention design. It confirmed that combining intervention with active nutrition and FITT-based exercise can effectively improve the L3SMI and reduce the incidence of chemotherapy-related neutropenia in patients with gastric cancer receiving SOX/XELOX chemotherapy. As the study was affected by the global epidemic of novel coronavirus pneumonia, the baseline L3SMI of the patients enrolled in the early stage of the study was higher than that of the patients enrolled in the later stage who received active nutrition combined with exercise intervention. A possible reason could be that the study area was closer to large specialized cancer hospitals in Beijing, China. Patients with better physical conditions and in earlier stages of cancer are more likely to seek treatment in superior, specialized hospitals after the epidemic. The baseline conditions of the patients treated at local hospitals were relatively poor, which was in line with the current medical conditions in the Ordos region. The lower SMI of the intervention group in the baseline period undoubtedly made the intervention more difficult, which theoretically would make the positive results of this experiment more convincing. We conducted a sensitivity analysis to examine the stability of the results (32).

In our previous study on L3SMI with active nutrition intervention, we showed that 57.9% of patients with gastrointestinal tumors in the active nutrition intervention group maintained stable or increased SMI. In addition, the reduction of L3SMI could be decreased but not reversed (-1.41% ± 8.49%) (14). A meta-analysis confirmed that anti-resistance exercise and a combination of anti-resistance with balance or cardiorespiratory exercise are the most effective interventions for improving sarcopenia in older patients, irrespective of whether nutritional intervention was combined (33). Hence, it can be suggested that combined intervention comprising exercise along with active nutrition can further improve the level of L3SMI in patients with gastric cancer. Skeletal muscle mass is determined by the balance between protein synthesis and degradation. cardiorespiratory combined with resistance exercise promotes protein synthesis, inhibited its degradation by regulating inflammation, autophagy mediators, and the ubiquitin-proteasome system (34). Furthermore, Exercise and nutrition interventions improve mitochondrial function, promote protein synthesis through Akt-mTORC1 and PGC-1α pathways in skeletal muscle (35). Therefore, this study investigating the effects of combined nutritional and exercise interventions on skeletal muscle index aligns with the aforementioned mechanisms (34, 35).

Studies have shown that moderate physical exercise during the treatment and postoperative rehabilitation is safe and patients can tolerate it well (36). Yamamoto et al. studied the effects of the nutrition and exercise combination therapy in ≥65-year

-old patients with gastric cancer and sarcopenia. Exercise training for an average period of 16 days significantly improved the index of grip strength (20.0 kg vs. 21.2 kg, *P* = 0.022) and reversed sarcopenia in 18.2% of patients, supporting the conclusion of the present study (37). Yamamoto et al. measured sarcopenia through BIA. Although there was improvement, it was not statistically significant. In the present study, we used CT to measure the L3SMI and obtained statistically significant results. Prof. Hall conducted a systematic review on a combination therapy comprising nutrition and exercise interventions for outpatients with advanced cancer. This systematic review included eight eligible studies, only two of which were prospective randomized controlled trials, totaling 99 patients. The primary endpoints of all studies focused on improvements in depression and physical endurance. Our research utilized CT-measured L3SMI as the main outcome indicator, which aligns with the evolving trends in sarcopenia management (30).

Kurk et al. conducted a phase III randomized controlled study (CAIRO3) on advanced colon cancer chemotherapy and revealed that skeletal muscle mass (SMM) decreased during the initial six cycles of CAPOX-BEV chemotherapy. However, the SMM improved during the subsequent maintenance therapy or observation periods, only to decline again upon disease progression when reintensified treatment was administered. This pattern aligns with the observed decrease in the SMI during chemotherapy in the control group of this study. Additionally, cancer patients undergoing systemic chemotherapy can still show improved SMM, thereby indirectly supporting the conclusion of our experimental intervention group, where combined nutritional and exercise management mitigated the decline in the L3SMI (38). Koya et al. conducted a study on 209 patients with liver cancer undergoing transcatheter arterial chemoembolization (TACE) and compared the changes in SMI (ΔSMI) between an intervention group (receiving exercise interventions combined with nutritional care based on Japanese nutritional management guidelines for cirrhosis) and a control group. The results showed that ΔSMI was significantly higher in the intervention group than it was in the control group (0.28 cm²/m² vs. -1.11 cm²/m², *P* = 0.0029) (39). Hence, it can be suggested that the combined nutritional and exercise intervention has a positive effect on maintaining or increasing the SMI, which also aligns with the conclusions of our study (39). However, Koya et al. studied active exercise and nutritional guidance intervention during chemoembolization in the hospital; the median time to evaluate SMI by CT was 50 days. In contrast, in our study, the patients received ambulatory chemotherapy and whole-course management of nutrition along with exercise intervention outside the hospital, with a much longer evaluation time of 12 weeks (39).

Nishida et al. conducted a one-arm clinical study on 18 older patients with gastric cancer who received exercise and branched-chain amino acid (BCAA) nutritional supplements after surgery. The patients were advised to walk over 5000 steps daily and perform resistance training comprising three sets of 10 repetitions of heel raises and squats. Throughout the intervention, dietitians provided personalized nutritional counselling to the patients. The average decrease in the SMI, as determined by using a body composition analyzer, was 4.6% at 1 week and 2.1% at 1 month. This study confirmed that the nutrition and exercise combined intervention program can effectively improve the degree of postoperative SMI decline in older patients with gastric cancer. This result is consistent with that of our study (40).

Our study confirmed that active nutrition combined with exercise intervention can significantly improve the reduction of L3SMI in patients with gastric cancer treated with SOX/XELOX chemotherapy. This improvement translated into better preservation of LBM in the intervention group compared to that in the control group. In addition, 64.1% of patients in the intervention group maintained or increased SMI, while only 31.6% of patients in the control group could do so. This improvement led to significant differences in the magnitude of changes in the BMI and BSA of the two groups, reaffirming the importance of dynamic and comprehensive management of SMI during chemotherapy (14). In our study, the incidence of grade ≥3 neutropenia in the intervention group was significantly lower than that in the control group. This finding is consistent with the recent research conducted by our team on the relationship between L3SMI and chemotherapy-induced toxicity; regardless of the baseline status or post-chemotherapy follow-up, the sarcopenia group exhibited a markedly higher incidence of grade ≥3 neutropenia than that exhibited by the non

-sarcopenia group. Additionally, the muscle-loss group demonstrated a more pronounced increase in toxic side effects relative to the muscle maintenance/gain group (6, 14, 41, 42). Williams et al. reviewed relevant literature and confirmed an inverse relationship between chemotherapy toxicity and muscle mass. Reduced muscle mass led to a smaller drug distribution volume, resulting in higher drug concentrations and increased toxicity (43). Hence, it is highly important to maintain muscle mass and quality through nutritional and exercise interventions during chemotherapy. The present study also found that patients with gastric cancer receiving adjuvant therapy had a significantly lower incidence of baseline grade ≥3 neutropenia compared to those receiving palliative therapy (*P* = 0.0308), while the adjuvant therapy group showed a higher baseline L3SMI than the palliative therapy group (33.36 ± 8.43 vs. 31.06 ± 6.96). These findings further suggest an inverse relationship between chemotherapy toxicity and muscle quality.

This study had several limitations too. First, it did not include quality-of-life assessment indicators for cancer patients. The impact of active nutrition combined with exercise intervention on the quality of life of patients should be evaluated with standardized scale systems, such as EORTC QLQ-C30. Secondly, the sample size was small, and because of the impact of COVID-19, a randomized intervention method could not be implemented. Third, no quantitative analysis of body fat measured by CT was conducted. Sarcopenic obesity, a poor prognostic indicator, has attracted much attention in recent years (44).

## 5 Conclusion and future prospects

This study, based on a prospective intervention design, confirmed that active nutrition and exercise combined intervention (based on the FITT model and five-step nutrition program) effectively improved the L3SMI in patients with gastric cancer undergoing SOX/XELOX chemotherapy and significantly reduced the incidence of grade ≥3 neutropenia. These findings suggest the feasibility and effectiveness of integrated nutrition and exercise interventions in sarcopenia. Future prospective randomized controlled trials should compare the effects of an integrated approach combining nutrition, exercise, and anti-inflammatory drugs on lean soft tissue and adipose tissue in cancer patients undergoing chemotherapy. They should also explore the development of stable and feasible nutrition exercise intervention methods suitable for cancer patients. In addition, they should attempt to develop patient

-reported outcome models based on AI technology platforms for a comprehensive management of changes in LBM and soft tissue in patients with cancer during the entire treatment process. Finally, the objective quantitative monitoring of motion should be realized through wearable devices in the future to further improve the stability of research conclusions. The combined nutrition and exercise intervention requires not only active implementation but also sustained adherence to achieve improvements in muscle mass and quality. For future studies, we recommend adopting dedicated sarcopenia assessment software to dynamically monitor the L3 skeletal muscle index (L3 SMI) using follow-up CT data from patients.

## Data Availability

The original contributions presented in the study are included in the article/**Supplementary Material**. Further inquiries can be directed to the corresponding authors.
